# Downregulation of interleukin-6 and C-reactive protein underlies a novel inhibitory role of microRNA-136-5p in acute lower extremity deep vein thrombosis

**DOI:** 10.18632/aging.103140

**Published:** 2020-11-14

**Authors:** Minghui Ou, Shaobo Hao, Jing Chen, Shibo Zhao, Shichao Cui, Jie Tu

**Affiliations:** 1 Department of Vascular Surgery, Qingdao Municipal Hospital, Qingdao 266011, P.R. China; 2 Department of Emergency, Qingdao Municipal Hospital, Qingdao 266011, P.R. China; 3 Department of Science and Education, Qingdao Municipal Hospital, Qingdao 266011, P.R. China

**Keywords:** acute lower extremity deep vein thrombosis, microRNA-136-5p, interleukin-6

## Abstract

Deep vein thrombosis (DVT) comprises a critical and common health condition with high incidence, mortality, and long-term adverse sequelae. Several differentially expressed microRNAs (miRNAs) have emerged as promising prognostic markers in DVT. The present study intended to explore the functional relevance of miR-136-5p in acute lower extremity DVT (LEDVT). Rat models of acute LEDVT were established and miR-136-5p expression was altered by agomir or antagomir to assess its effects. In addition, *in vitro* gain- and loss-experiments, prior to exposure to CoCl_2_, were performed to investigate effects of miR-136-5p on human umbilical vein endothelial cell (HUVEC) apoptosis and levels of interleukin-6 (IL-6) and C-reactive protein (CRP). miR-136-5p was downregulated, whereas IL-6 and CRP were elevated in acute LEDVT patients. Notably, miR-136-5p was confirmed to target both IL-6 and CRP. Overexpression of miR-136-5p led to reduced length, weight, and ratio of weight to length of the venous thrombus. Furthermore, overexpressed miR-136-5p downregulated the expression of IL-6 and CRP, consequently inhibiting HUVEC apoptosis. Conjointly, our data indicate that the overexpression of miR-136-5p has the potential to bind to the 3’-UTR in the mRNAs for IL-6 and CRP and mitigate acute LEDVT, which provides a basis for new therapeutic targets in acute LEDVT treatment.

## INTRODUCTION

Venous thromboembolism (VTE) is recognized as the third most common cardiovascular disorder and includes deep-vein thrombosis (DVT) and pulmonary embolism (PE) [1, 2]. DVT involves the formation of a blood clot in a deep vein, and frequently occurs in the lower extremities. As a matter of concern, DVT remains a significant cause of morbidity and mortality all over the world [3]. Specifically, acute lower extremity deep vein thrombosis (LEDVT) is a serious medical disorder that can result in mortality or major disability due to PE [4]. The risk factors of acute LEDVT in adults include inferior vena cava (IVC) abnormalities, which are typically caused by either atresia or chronic thrombosis [5, 6]. Acute LEDVT may potentially lead to multiple complications such as thrombosis recurrence, PE, and syndrome post thrombosis [7]. Patients suffering from initial-stage LEDVT can be diagnosed with precision using an imaging approach, compression ultrasonography; these are both simple and non-invasive [8]. Among the treatment measures for acute LEDVT, it has been reported that catheter-directed thrombolysis (CDT) by directly infusing thrombus agent serves as an alternative choice to standard anticoagulant therapy with an acceptable complication rate and decreased incidence of post-thrombotic syndrome (PTS) [9, 10]. However, CDT entails drawbacks in the treatment of acute LEDVT such as pain, risk of bleeding, and prolonged hospitalization [11, 12]. Therefore, the importance of developing new therapeutic approaches for the treatment of acute LEDVT cannot be overestimated.

microRNAs (miRNAs) are a family of short endogenous noncoding RNAs which play significant roles in the regulation of gene expression by downregulating target genes of mRNAs or the suppression of protein translation [13]. The differential expression of several miRNAs in vein thrombosis (VT) has been well explored [14]. For example, miR-136-5p is reported to have a negative correlation with the presence of DVT [15]. Notably, Zhang et al. have reported that miR-136 is involved in the apoptosis of human umbilical vein endothelial cells (HUVECs) induced by hypoxia [16]. It has been demonstrated that interleukin-6 (IL-6) and C-reactive protein (CRP) are both pro-inflammatory markers associated with VTE, and lowering of IL-6 can alleviate PTS after DVT [17]. In addition, neutralizing IL-6 during VT has been found to reduce the intimal thickness and fibrosis of the vein wall, thus acting as an underlying marker to protect against fibrotic complication in PTS [18]. CRP is known as a protein that is typically associated with the acute phase that is found increased in many chronic and acute inflammatory diseases [19]. In particular, CRP expression is found to be up-regulated in DVT patients and has been considered as an important predictor of the risk for recurrent VT [20]. Based on existing evidence, we hypothesized that miR-136-5p may regulate IL-6 and CRP to play a functional role in acute LEDVT. Therefore, the present study investigated the potential mechanisms of miR-136-5p involvement in acute LEDVT by applying *in vitro* and *in vivo* experiments.

## RESULTS

### Poor expression of miR-136-5p and abundant expression of IL-6 and CRP in DVT patients

**Figure 1:**
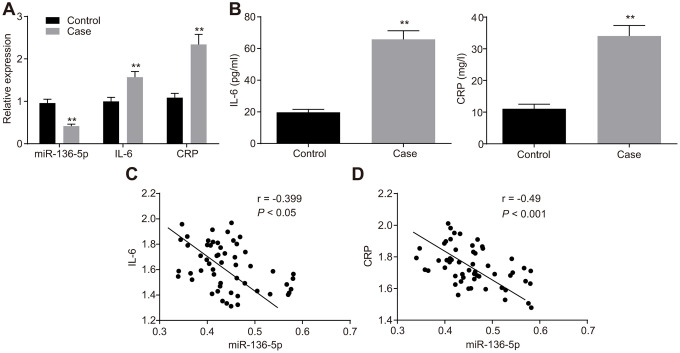
**miR-136-5p is downregulated, but IL-6 and CRP are upregulated in DVT patients.** (**A**) The expression levels of miR-136-5p, IL-6, and CRP in patients with (n = 55) and without DVT (n = 74) detected by RT-qPCR. (**B**) The protein expression levels of miR-136-5p, IL-6, and CRP in patients with (n = 55) and without DVT (n = 74) detected by ELISA. (**C**) The association between miR-136-5p expression and IL-6 mRNA expression analyzed by Pearson’s correlation analysis. (**D**) The association between miR-136-5p expression and CRP mRNA expression analyzed by Pearson’s correlation analysis. ** *p* < 0.01 compared with the patients without DVT. Measurement data were expressed as mean ± standard deviation. Data from two groups were compared using independent sample *t*-test. miR-136-5p, microRNA-136-5p; IL-6, interleukin-6; CRP, C-reactive protein; DVT, deep vein thrombosis; RT-qPCR, reverse transcription quantitative polymerase chain reaction; ELISA, enzyme linked immunosorbent assay.

The expression of miR-136-5p, and mRNA and protein expression levels of IL-6 and CRP in peripheral blood of patients with and without DVT were evaluated using reverse transcription quantitative polymerase chain reaction (RT-qPCR) (Figure 1A) and enzyme linked immunosorbent assay (ELISA) (Figure 1B). The results showed that patients with DVT typically exhibited lower levels of miR-136-5p but higher mRNA and protein levels of IL-6 and CRP than patients without DVT. Furthermore, Pearson’s correlation analysis was employed to test the correlation between miR-136-5p and IL-6 mRNA expression levels and that between miR-136-5p and CRP mRNA expression levels. The results illustrated that miR-136-5p was negatively correlated with both IL-6 (r = -0.399; *p* < 0.05) and CRP (r = -0.490, *p* < 0.001) (Figure 1C, 1D). These results evidenced low miR-136-5p expression levels with highly expressed IL-6 and CRP levels in patients with DVT, where miR-136-5p had a negative association with both IL-6 and CRP.

### Both IL-6 and CRP are target genes of miR-136-5p

**Figure 2:**
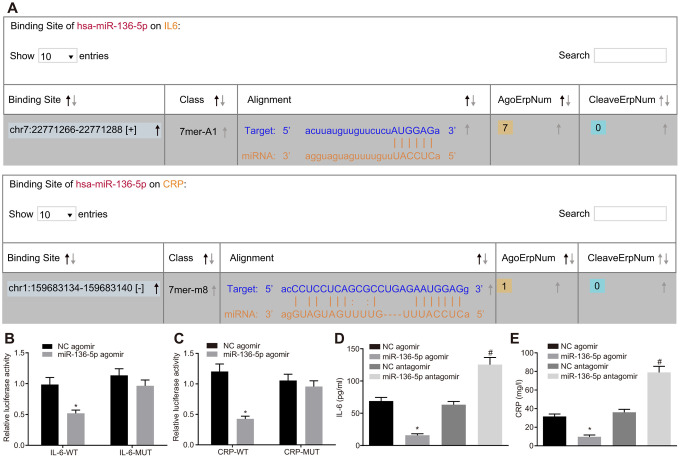
**miR-136-5p directly binds to IL-6 and CRP and downregulates their expression.** (**A**) The binding sites between miR-136-5p and IL-6 as well as between miR-136-5p and CRP predicted by using a web-based bioinformatic prediction resource. (**B**) The interaction between miR-136-5p and IL-6 verified by dual-luciferase reporter gene assay. (**C**) The interaction between miR-136-5p and CRP verified by dual-luciferase reporter gene assay. (**D**) The level of IL-6 in the culture medium supernatant of HUVECs after alteration of miR-136-5p expression, as detected by ELISA. (**E**) The level of CRP in the culture medium supernatant of HUVECs after alteration of miR-136-5p, as detected by ELISA. * *p* < 0.05 *vs*. treatment of NC agomir; # *p* < 0.05 *vs*. treatment of NC antagomir. Measurement data were expressed as mean ± standard deviation. Data from two groups were compared using independent sample *t*-test. Each experiment was repeated three times. miR-136-5p, microRNA-136-5p; IL-6, interleukin-6; CRP, C-reactive protein; ELISA, enzyme linked immunosorbent assay; HUVECs, human umbilical vein endothelial cells; ANOVA, analysis of variance.

Using the web-based bioinformatic resource ‘starBase’ the existence of binding sites between miR-136-5p and IL-6 as well as between miR-136-5p and CRP was predicted (Figure 2A). The recombinant plasmid of the luciferase reporter gene was obtained through the insertion of IL-6 mRNA 3’untranslated region (UTR) or CRP mRNA 3’UTR in order to verify the status of their interaction. Dual-luciferase reporter gene assay results displayed that luciferase activities of IL-6-wild type (Wt) and CRP-Wt were relatively lower (*p* < 0.05) while those of IL-6-Mutant (Mut) and CRP-Mut did not differ significantly following treatment with miR-136-5p agomir (*p* > 0.05) (Figure 2B, 2C). In addition, ELISA results indicated that levels of IL-6 and CRP were coherently reduced in the culture medium supernatant of HUVECs treated with miR-136-5p agomir. However, levels of IL-6 and CRP were significantly increased in cells treated with miR-136-5p antagomir (Figure 2D, 2E). In sum, these results showed that miR-136-5p targeted IL-6 and CRP and downregulated their expression.

### Elevation of miR-136-5p impedes progression of acute LEDVT

**Figure 3:**
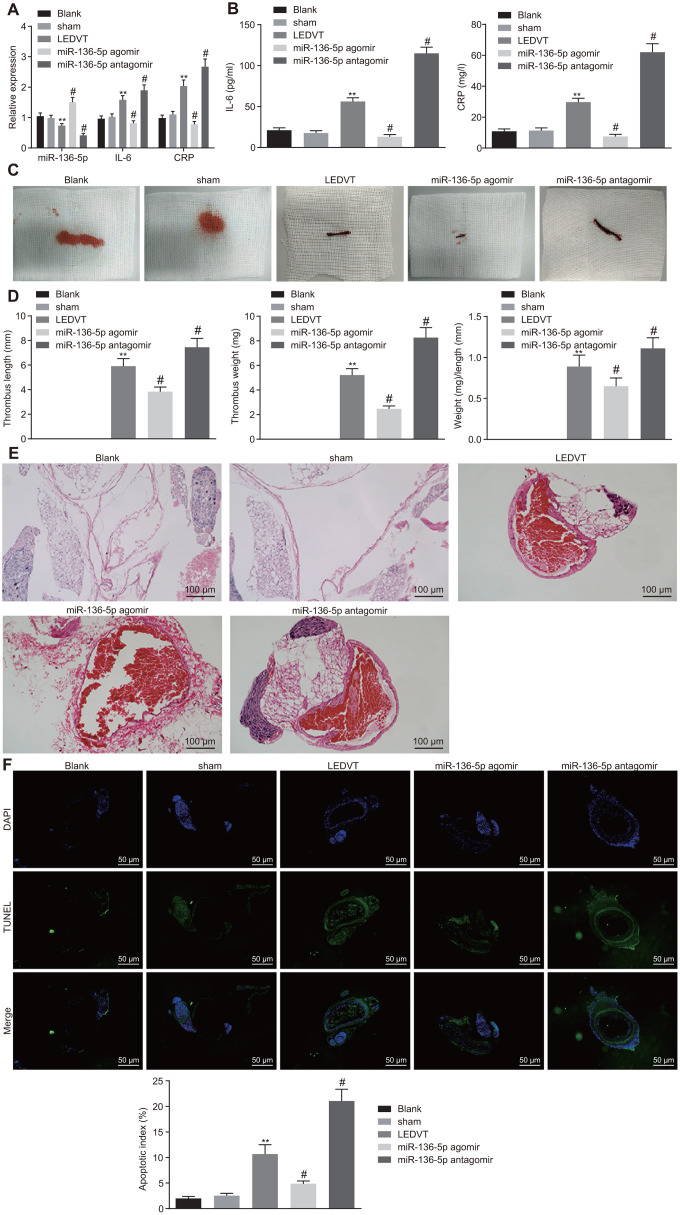
**Acute LEDVT is mitigated by the upregulation of miR-136-5p.** The rats used for following assays included rats injected with normal saline alone, sham-operated rats, acute LEDVT model rats, and rats treated with miR-136-5p agomir or miR-136-5p antagomir. (**A**) The expression levels of miR-136-5p, IL-6 and CRP in the vein tissues of rats determined by RT-qPCR. (**B**) The protein expression levels of IL-6 and CRP in peripheral blood serum of rats determined by ELISA. (**C**) Images of acute LEDVT in rats. (**D**) The length, weight, and the ratio of weight to length of the venous thrombus in rats. (**E**) The pathological changes in vein tissues of rats determined by HE staining (100 ×). (**F**) The apoptosis of endothelial cells in the femoral vein in rats measured by TUNEL assay (200 ×). ** *p* < 0.01 compared with sham-operated rats; # *p* < 0.05 compared with the acute LEDVT model rats. Measurement data were expressed as mean ± standard deviation. Data from multiple groups were compared using one-way ANOVA. N = 12 for rats in each group. LEDVT, lower extremity deep vein thrombosis; miR-136-5p, microRNA-136-5p; IL-6, interleukin-6; CRP, C-reactive protein; RT-qPCR, reverse transcription quantitative polymerase chain reaction; ELISA, enzyme linked immunosorbent assay; HE, hematoxylin-eosin; TUNEL, terminal deoxynucleotidyl transferase-mediated dUTP-biotin nick end labeling; ANOVA, analysis of variance.

Following the determination of the correlation of miR-136-5p with IL-6 and CRP, the focus of the study was shifted to the effects of miR-136-5p on acute LEDVT. The results of RT-qPCR, which were used to evaluate the expression of miR-136-5p, IL-6, and CRP in vein tissues, revealed that the expression levels of miR-136-5p, IL-6, and CRP in vein tissues did not fluctuate greatly in rats injected with normal saline alone and in sham-operated rats. In comparison to the rats that underwent acute LEDVT modeling, the expression levels of miR-136-5p were significantly higher while those of IL-6 and CRP were remarkably lower in the rats injected with miR-136-5p agomir. The opposite results were noted in rats injected with miR-136-5p antagomir (Figure 3A). The ELISA assays displayed that the levels of IL-6 and CRP were markedly downregulated in miR-136-5p agomir-treated rats but were significantly upregulated in miR-136-5p antagomir-treated rats (Figure 3B). In addition, rats injected with normal saline alone and sham-operated rats showed IVC walls that were unobstructed. Moreover, in these rats, the bloodstream was visible and apparent inside the vascular lumen, and the vessel diameters were uniform with thin, soft, and elastic vessel walls and without any evident thrombus in vessels. However, in rats that underwent acute LEDVT modeling and in rats injected with miR-136-5p agomir or miR-136-5p antagomir, the IVC presented different extremes of edema and venous walls were seen as dark purple with venous thrombus (Figure 3C). The length and weight of the venous thrombus were considered fundamental indices for the purpose of quantifying the degree of venous thrombosis. As illustrated in Figure 3D, no venous thrombus was observed in rats injected with normal saline alone and sham-operated rats. When compared with the acute LEDVT model rats, the length, weight, and the ratio of weight to length of the venous thrombus were found significantly reduced in rats treated with miR-136-5p agomir (*p* < 0.05), but significantly elevated in rats treated with miR-136-5p antagomir (*p* < 0.05), implying that the upregulation of miR-136-5p had the ability to decrease the length and weight of the venous thrombus. Hematoxylin-eosin (HE) staining was conducted for pathological analysis of the vein tissues and the results revealed no venous thrombus in rats solely injected with normal saline and sham-operated rats. However, incomplete venous thrombus was observed in miR-136-5p agomir-treated rats, and complete venous thrombus was noted in both miR-136-5p antagomir-treated rats and acute LEDVT model rats (Figure 3E). Following this section of the experiment, terminal deoxynucleotidyl transferase-mediated dUTP-biotin nick end labeling (TUNEL) assay was performed in order to detect the effects of miR-136-5p on the apoptosis of endothelial cells in the femoral vein. The results indicated that endothelial cell apoptosis did not differ greatly in rats treated solely with normal saline and sham-operated rats. In contrast to the acute LEDVT model rats, the rats treated with miR-136-5p agomir demonstrated significantly attenuated endothelial cell apoptosis whereas rats treated with miR-136-5p antagomir presented significantly enhanced endothelial cell apoptosis (*p* < 0.05) (Figure 3F). These results supported the notion that the upregulation of miR-136-5p could possibly inhibit the progression of acute LEDVT.

### IL-6 and CRP reverse the protective effects of upregulated miR-136-5p against acute LEDVT

**Figure 4:**
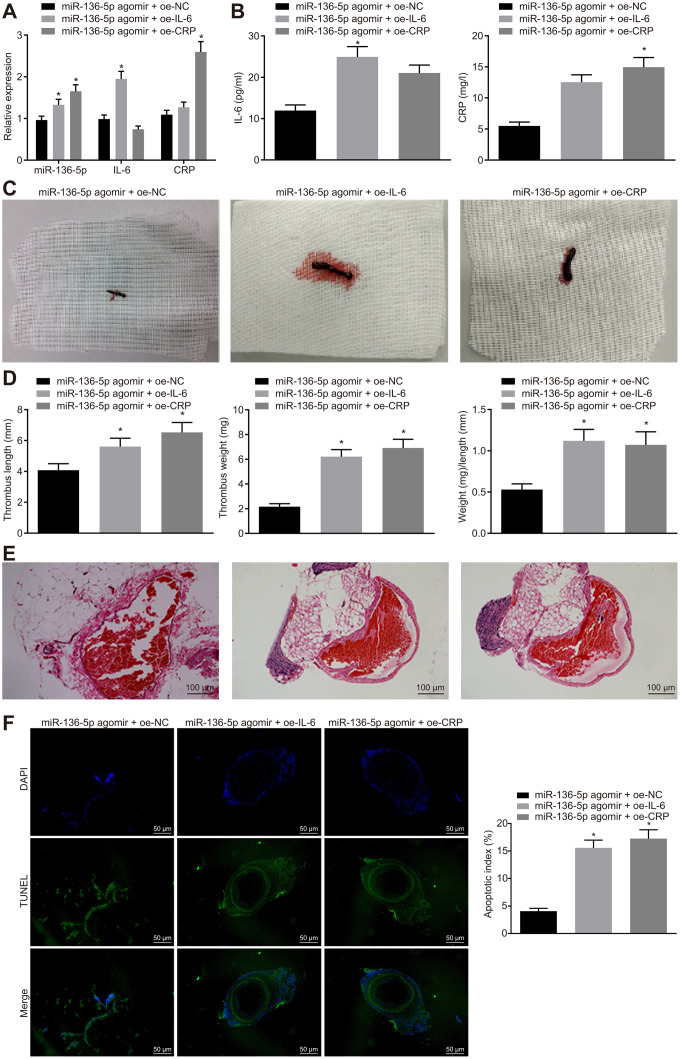
**The protective role of miR-136-5p elevation against acute LEDVT is reversed by IL-6 and CRP.** The rats used for following assessments were rats treated with miR-136-5p agomir + oe-IL-6, oe-CRP, or oe-NC. (**A**) The expression levels of miR-136-5p, IL-6, and CRP in vein tissues of rats determined by RT-qPCR. (**B**) The protein expression of IL-6 and CRP in peripheral blood serum of rats determined by ELISA. (**C**) The images of acute LEDVT in rats. (**D**) The length, weight, and the ratio of weight to length of the venous thrombus in response to miR-136-5p agomir + oe-IL-6, oe-CRP, or oe-NC. (**E**) Pathological changes in vein tissues in rats assessed by HE staining (100 ×). (**F**) The apoptosis of endothelial cells in the femoral vein in rats assessed by TUNEL assay (200 ×). * *p* < 0.05 compared with the rats treated with combined treatment of miR-136-5p agomir and oe-NC. Measurement data were expressed as mean ± standard deviation. Data from multiple groups were compared using one-way ANOVA. N = 12 for rats in each group. LEDVT, lower extremity deep vein thrombosis; miR-136-5p, microRNA-136-5p; IL-6, interleukin-6; CRP, C-reactive protein; RT-qPCR, reverse transcription quantitative polymerase chain reaction; ELISA, enzyme linked immunosorbent assay; HE, hematoxylin-eosin; TUNEL, terminal deoxynucleotidyl transferase-mediated dUTP-biotin nick end labeling; ANOVA, analysis of variance; NC, negative control.

Thereafter, a series of assays were employed to investigate the regulatory mechanisms involving IL-6 and CRP in miR-136-5p effects on acute LEDVT in the different treatment groups of rats. Initially, mRNA and protein expression levels of IL-6 and CRP in rat vein tissues were detected using RT-qPCR (Figure 4A) and ELISA (Figure 4B). The results indicated that compared with the rats treated with both miR-136-5p agomir and overexpression vector negative control (oe-NC), the mRNA and protein expression levels of IL-6 were significantly higher in rats treated with both miR-136-5p agomir and oe-IL-6, and similarly, CRP levels were also clearly elevated in rats treated with both miR-136-5p agomir and oe-CRP. However, considering the changes of acute LEDVT in rats after different treatments, it was noted that the length, weight, and the ratio of weight to length of the venous thrombus were elevated following treatments with combination of miR-136-5p agomir and oe-IL-6 or combination of miR-136-5p agomir and oe-CRP in comparison to that with the combination of miR-136-5p agomir and oe-NC (all *p* < 0.05; Figure 4C, 4D). Subsequently, pathological changes in vein tissues in rats that underwent different treatments were analyzed using HE staining. Combined treatment of miR-136-5p agomir and oe-IL-6 or combined treatment of miR-136-5p agomir and oe-CRP led to complete venous thrombosis versus the combined treatment of miR-136-5p agomir and oe-NC (Figure 4E). The TUNEL assay for endothelial cell apoptosis detection showed that the apoptosis of endothelial cells was markedly enhanced in response to the combined treatment of miR-136-5p agomir and oe-IL-6 or the combined treatment of miR-136-5p agomir and oe-CRP in contrast to the combined treatment of miR-136-5p agomir and oe-NC (*p* < 0.05) (Figure 4F). These results supported a conclusion that IL-6 and CRP could reverse the mitigative effects of miR-136-5p elevation on acute LEDVT.

### miR-136-5p targets IL-6 and CRP to inhibit the apoptosis of HUVECs *in vitro*

**Figure 5:**
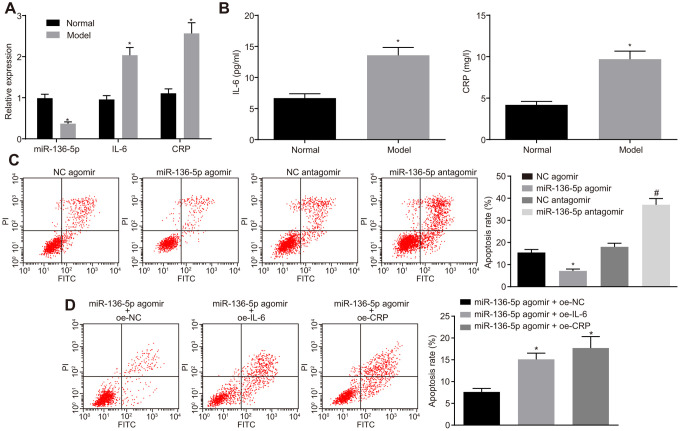
**The suppressive effect of miR-136-5p elevation on HUVEC apoptosis *in vitro* is reversed by IL-6 and CRP.** (**A**) The expression levels of miR-136-5p, IL-6, and CRP in normal and HUVECs exposed to CoCl_2_ determined by RT-qPCR (* *p* < 0.05 compared with normal HUVECs). (**B**) The levels of IL-6 and CRP in the culture medium supernatant of normal and HUVECs exposed to CoCl_2_ determined by ELISA (* *p* < 0.05 compared with normal HUVECs). (**C**) Apoptosis rate of HUVECs following the treatment of miR-136-5p agomir or miR-136-5p antagomir measured using flow cytometry (* *p* < 0.05 compared with HUVECs treated with NC agomir; # *p* < 0.05 compared with HUVECs treated with NC antagomir). (**D**) The apoptosis rate of HUVECs after treatment of miR-136-5p agomir + oe-IL-6, oe-CRP, or oe-NC detected by rescue experiment (* *p* < 0.05 compared with HUVECs treated with miR-136-5p agomir and oe-NC). Measurement data were expressed as mean ± standard deviation. Data from two groups were compared using independent sample *t*-test and data from multiple groups using one-way ANOVA. Each experiment was repeated three times. miR-136-5p, microRNA-136-5p; IL-6, interleukin-6; CRP, C-reactive protein; RT-qPCR, reverse transcription quantitative polymerase chain reaction; ELISA, enzyme linked immunosorbent assay; HUVECs, human umbilical vein endothelial cells; NC, negative control; ANOVA, analysis of variance; CoCl_2_, cobalt chloride.

After determination of the reverse effects of IL-6 and CRP on the protective role of miR-136-5p, the study was refocused to investigate the role of miR-136-5p in HUVECs. For this purpose, HUVECs were exposed to 250 μM CoCl_2_ for 12 hours to simulate hypoxic/ischemic conditions and induce a hypoxia damage model. RT-qPCR was performed to measure the expression levels of miR-136-5p, IL-6 and CRP in HUVECs and results indicated that the expression levels of miR-136-5p were notably downregulated whereas those of IL-6 and CRP were evidently upregulated in HUVECs upon exposure to CoCl_2_ (Figure 5A). In agreement, ELISA results indicated that the culture medium supernatant of HUVECs exposed to CoCl_2_ showed an increase in the levels of IL-6 and CRP (Figure 5B). Prior to exposure to CoCl_2,_ HUVECs were treated with miR-136-5p agomir or miR-136-5p antagomir. Flow cytometry showed that the apoptosis rate of HUVECs was significantly lower in response to miR-136-5p agomir and markedly increased in response to miR-136-5p antagomir (Figure 5C). Furthermore, the inhibitory effect that miR-136-5p overexpression on HUVECs apoptosis could be countered by IL-6 and CRP (Figure 5D). Overall, these findings suggested that miR-136 bound to IL-6 and CRP, leading to the suppression of HUVEC apoptosis *in vitro*.

## DISCUSSION

Acute LEDVT is commonly caused by obstruction in large veins due to thrombus formation, which is usually associated with severe pain and swelling [21]. LEDVT is a critical disease with a high mortality rate due to PE or PTS [22]. Emerging evidence has highlighted that miRNAs may comprise novel potential biomarkers and therapeutic targets in DVT owing to their mechanistic roles [23]. The current study demonstrated that overexpression of miR-136-5p could alleviate acute LEDVT by targeting IL-6 and CRP.

An increasing number of miRNAs have been documented as poorly expressed in LEDVT animals or patients, such as miR-26a [24]. In the current study, miR-136-5p was found to be poorly expressed, whereas IL-6 and CRP were highly expressed in acute LEDVT patients. Consistent with the findings of the present study, Wang et al. previously demonstrated that the plasma level of miR-136-5p is significantly decreased in DVT patients as compared to patients without DVT [15]. Brandon et al. indicated that IL-6 was elevated in VT and suggested that IL-6 depletion could be a novel biomarker for PTS [18]. Elevation of IL-6 is shown to increase the risk of DVT and related complications [25].

In addition, both IL-6 and high-sensitive CRP are well documented as highly expressed in acute DVT [26, 27]. It is established that high-sensitivity CRP is an inflammatory cytokine associated with thrombosis [28]. In patients with DVT, the elevation of pro-inflammatory markers, including IL-6 and CRP has been shown to be caused by VT, rather than being its precursor [29].

Furthermore, this study demonstrated that overexpression of miR-136-5p had the potential to inhibit the apoptosis of HUVECs and alleviate acute LEDVT by negatively regulating both IL-6 and CRP. HUVECs are extensively employed to establish *in vitro* experimental models for exploring blood vessel endothelial cells [30]. Apoptosis of HUVECs is documented as a mechanism involved in diverse cardiovascular diseases, including thrombus formation, and can trigger the dysfunction of endothelium and other complications associated with vascular diseases [16, 31]. The increase in apoptosis rate of neurons in rats with spinal cord ischemic injury is shown to be salvaged by increased expression of miR-136 [32]. Similar to our results, IL-17, another proinflammatory cytokine, was found to trigger apoptosis of vascular endothelial cells, while it positively correlated with IL-6 [33], indicating that IL-6 may be implicated in the induction of the vascular endothelial cell apoptosis. However, a targeting relationship between miR-136-5p and IL-6 or CRP has never been explored before. Notably, this study uncovered that miR-136-5p could bind to 3’UTR of both IL-6 mRNA and CRP mRNA and then negatively regulate their expression in acute LEDVT. Decreased miR-136-5p level can help inhibit the inflammatory response in oxygen glucose deprivation/reperfusion (OGD/R)-induced damage [34] and IL-6 is a well-studied proinflammatory cytokine [35], suggesting a negative correlation of miR-136-5p with IL-6. In addition, CRP is an inflammatory marker and CRP levels are enhanced by proinflammatory cytokines such as IL-6 and IL-1 in the liver [36]. Thus, it can be reasoned that miR-136-5p negatively regulates CRP expression. Furthermore, CRP has been reported to be an important mechanistic link between inflammation and thrombosis, since increased CRP expression enhances the thrombotic response to vascular injury and inflammation upregulates CRP expression *in vivo* [37]. In addition, CRP is found capable of enhancing the activation of the coagulation cascade and potentiating an inflammatory response by dissociating into monomeric form in case of antineutrophil cytoplasmic antibody-associated vasculitis [38]. These findings demonstrated the involvement of the miR-136-5p/IL-6/CRP axis in the progression of acute LEDVT.

**Figure 6:**
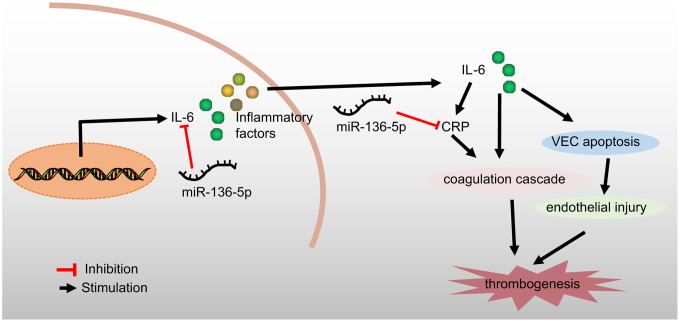
**A schematic diagram depicting the regulatory mechanism of miR-136-5p acting through IL-6 and CRP in acute LEDVT.** Overexpression of miR-136-5p targeted IL-6 and CRP to block the coagulation cascade and consequent endothelial injury, thus inhibiting venous thrombus formation and ultimately alleviating acute LEDVT. miR-136-5p, microRNA-136-5p; IL-6, interleukin-6; CRP, C-reactive protein; LEDVT, lower extremity deep vein thrombosis.

In conclusion, the current study demonstrated that overexpression of miR-136-5p could bind to and negatively regulate both IL-6 and CRP to ameliorate the development of acute LEDVT (Figure 6). This study provides a theoretical basis for a deeper understanding of the mechanisms underlying acute LEDVT, thus enabling the development of new therapeutic strategies for the prevention and treatment of this disease. However, in order to explore other miRNAs implicated in the development of acute LEDVT as well as the other possible target genes of miR-136-5p, further studies are essential and these may validate current findings to further the translational potential of this direction.

## MATERIALS AND METHODS

### Study subjects

A total of 55 patients diagnosed with DVT after hip and knee replacements, and 74 patients without DVT during the perioperative period at Qingdao Municipal Hospital from March 2016 to November 2017 were enrolled in this study. The 129 included patients were aged between 35 and 85 years, with an average age of 49.8 ± 10.4 years. Among these patients, 79 patients were males and 50 were females; 25 patients had a body mass index (BMI) of approximately over 25 kg/m^2^ (22.5%) and 104 patients had a BMI of less than 25 kg/m^2^ (77.5%); 38 patients experienced multiple fractures (29.5%), 39 patients had a femoral fracture (30.2%), and 52 patients had tibia and fibula fractures (40.3%). The patients were enrolled in this study if they met the following criteria: (1) patients diagnosed with DVT and confirmed to suffer from a fracture of the lower limbs through X-ray examination; (2) patients with a lower limb fracture who had no history of DVT; (3) patients with no pathological fractures or dysfunctions in heart, lung, liver, and kidney. Patients having malignant tumors or suffering from myeloproliferative diseases, common infection, severe autoimmune diseases, severe mental illnesses or liver dysfunction were omitted from the study. The disease course and DVT condition of the patients was closely analyzed during the entire study period [39].

### Clinical sample collection and index evaluation

A total of 2 mL peripheral blood was drawn while patients were on empty stomach in the morning on the day of treatment. The blood samples were added with anticoagulant heparin, and centrifuged at 8000 r/min at 4°C for 10 minutes to collect the plasma and red blood cells, followed by the extraction of RNA from total red cells. The expression levels of miR-136-5p, IL-6, and CRP in the cells were detected using RT-qPCR. The levels of IL-6 and CRP in plasma were measured using ELISA kits (Shanghai Bogu biotech, Co, Ltd, Shanghai, China) [39].

### RT-qPCR

Total RNA was extracted from the femoral vein tissues of the rats in each group using RNA extraction kits (Invitrogen, Carlsbad, CA, USA). The primers (Table 1 and Supplementary Table 1) for miR-136-5p, IL-6, CRP, U6, and β-actin were synthesized by Takara Biotech-nology Ltd. (Dalian, Liaoning, China). Subsequently, the extracted total RNA was reverse transcribed into complementary DNA (cDNA) using the PrimeScript reverse transcription kits. RT-qPCR for miR-136-5p was carried out using miRNA qPCR Quantitation Kit (Shanghai GenePharma Co., Ltd., Shanghai, China) [40] and that for IL-6 and CRP was performed using SYBR Premix Ex Taq II kit (Takara, Tokyo, Japan), in accordance with the manufacturer’s instructions. U6 was used as the internal control for miR-136-5p and β-actin was used for IL-6 and CRP assays. The expression levels of miR-136-5p, IL-6, and CRP were calculated by applying the 2^-ΔΔCt^ method.

**Table 1:** Primer sequences for RT-qPCR.

**Gene**	**Primer sequence**
miR-136-5p (Homo sapiens)	F: 5'-CGCGACTCCATTTGTTTTGA-3'
	R: 5'-AGTGCAGGGTCCGAGGTATT-3'
miR-136-5p (Rattus norvegicus)	F: 5'-ACUCCAUUUUGAUGAUGGA-3'
	R: 5'-CAUCAAAACAAAUGGAGUUU-3'
U6 (Homo sapiens)	F: 5'-CTCGCTTCGGCAGCACA-3'
	R: 5'-AACGCTTCACGAATTTGCGT-3'
U6 (Rattus norvegicus)	F: 5'-ATGACGTCTGCCTTGGAGAAC-3'
	R: 5'-TCAGTGTGCTACGGAGTTCAG-3'
IL-6 (Homo sapiens)	F: 5'-GGTACATCCTCGACGGCATCT-3'
	R: 5'-GTGCCTCTTTGCTGCTTTCAC-3'
IL-6 (Rattus norvegicus)	F: 5'-GATCGACCTGGAGACTTCACAGAGGATACC-3'
	R: 5'-GATCGACCATGGTTATATCCAGTTTGGAAGCATCC-3'
CRP (Homo sapiens)	F: 5'-ACCACAGTCCATGCCATCAC-3'
	R: 5'-CACCACCTTCTTGATGTCATC-3'
CRP (Rattus norvegicus)	F: 5'-GTAGGTGGGCCTGAAATACTGTTC-3'
	R: 5'-AAGCCAAAGCTCTACAATTCCTGT-3'
β-actin (Homo sapiens)	F: 5'-ATCATGTTTGAGACCTTCAACA-3'
	R: 5'-CATCTCTTGCTCGAAGTCCA-3'
β-actin (Rattus norvegicus)	F: 5'-ATGGATCCTGTGGCATCCA-3'
	R: 5'-CGCTCAGGAGGAGCAATGAT-3'

Note: RT-qPCR, reverse transcription quantitative polymerase chain reaction; F, forward; R, reverse; miR-136-5p, microRNA-136-5p; IL-6, interleukin-6; CRP, C-reactive protein.

### Dual-luciferase reporter gene assay

The target gene of miR-136-5p was predicted using the web-based bioinformatic resource (starBase). Dual-luciferase reporter gene assay was conducted in order to verify whether IL-6 and CRP were direct target genes of miR-136-5p. The pMIR-reporter plasmid (MLCC13738, Miaolingbio Inc., Wuhan, Hubei, China) was introduced using the endonuclease sites SpeI and Hind III. The Mut site of complementary sequence was designed on the IL-6-Wt and CRP-Wt. IL-6-Mut or CRP-Mut plasmid was constructed with promoter-Renilla luciferase reporter plasmid (PRL-TK; E2241, Promega Corporation, Madison, WI, USA) used as the internal reference. miR-136-5p mimic and miR-136-5p negative control (NC) were co-treated with luciferase reporter plasmid and then were transferred into human embryonic kidney (HEK)-293T cells (CRL1415, Shanghai Xin Yu Biotech Co., Ltd, Shanghai, China). Finally, the fluorescence density was detected using a fluorescence detector (Glomax20/20, Promega Corporation, Madison, WI, USA) [39].

### Experimental animals

A total of 96 specific-pathogen-free (SPF) grade Sprague Dawley (SD) rats weighing 250 ± 20 g (half males and half females) were purchased from Beijing Vital River Laboratory Animal Technology Co., Ltd. (Beijing China). The rats were housed adaptatively for 1 week at 18°C - 22°C with a humidity of 40% - 70%, natural sunlight, and a noise grade < 50 db, with free access to water and food. Following this, the rats were grouped as follows: rats intraperitoneally injected with 20 mL normal saline for 4 days without any other treatment, sham-operated rats (rats intraperitoneally injected with 20 mL normal saline for 4 days with the inner thigh skin incised to expose the femoral vein), acute LEDVT model rats (rats intraperitoneally injected with 20 mL normal saline for approximately 4 days to prepare the rat model of acute LEDVT), and rats intraperitoneally injected with 20 mL normal saline containing 400 pmol miR-136-5p agomir, 400 pmol miR-136-5p antagomir, 400 pmol miR-136-5p agomir + oe-NC, 400 pmol miR-136-5p agomir + oe-IL-6, or 400 pmol miR-136-5p agomir + oe-CRP 4 days before modeling. Both miR-136-5p agomir and miR-136-5p antagomir were purchased from RiboBio company (Guangzhou, Guangdong, China) [39, 40]. Lentiviral vectors (LV) oe-IL-6 and oe-CRP and their controls were obtained from Shanghai GenePharma Co., Ltd. (Shanghai, China).

### Establishment of rat models of acute LEDVT

A rat model of acute LEDVT was established with the both sides of femoral veins blocked aseptically using a vascular clamp. To begin with, the rats were anaesthetized by intraperitoneal injections of 3% pentobarbital sodium (1 mL/kg, Shanghai Xingzhi Chemical Plant, Shanghai, China). Then, the inner thighs of rats were shaved and the rats were fixed in a supine position. Next, the inner thigh skin was incised longitudinally with the femoral veins exposed 2 cm from the incision. The veins at three different positions were blocked with the mosquito clamp and the incision was sutured after modeling. The rats were regularly fed after recovery. One day after modeling, the swelling of the lower limb and acral skin color were noted by the naked eye. Rats with clearly swollen and contusive lower limbs were selected as the experimental animals [39, 41]. A total of 2 mL peripheral blood was extracted from the rats 24 hours after modeling. The blood, with the addition of anticoagulant heparin, was centrifuged at 2000 g and at 4°C for 20 minutes to collect the plasma. The levels of CRP and IL-6 in plasma were then measured using turbidimetry standard method and ELISA (R & D Systems Europe Ltd, Abingdon, UK), respectively [27].

### Length and weight of the venous thrombus

The rats were anaesthetized by intraperitoneal injection of 3% pentobarbital sodium (1 mL/kg) 24 hours after modeling and were disinfected in a supine position. The original incision was incised again approximately 2 - 3 cm along the abdominal cavity, layer by layer. The abdominal contents were lightly pushed out to the left side. IVC tissues were isolated and inflammatory hyperplasia of the operative field around the vein was cleaned up so that the vein tissues could be evidently seen in the model sections. The IVC and embolus were resected under ligature. Subsequently, the weight and length of the venous thrombus were measured and recorded. In rats only injected with 20 mL normal saline, the IVC tissues (about 1 - 1.5 cm) were dissected at the termination of the left renal vein, and the weight (SartoriusBSA224S-CW analytical balance, four decimal places) and length (Mitutoyo 530-119, Vernier, Caliper, two decimal places) of the venous thrombus were each measured and recorded. The ratio of thrombus weight to length was analyzed in rats with different treatments. The vessel wall and embolus were separated and stored at -80°C [39].

### HE staining

IVC tissues were fixed with 4% paraformaldehyde solution for 16 - 18 hours. Then, the tissues were dehydrated with gradient alcohol (70%, 80%, 90%, and 100%), paraffin-embedded and sliced into 4-μm sections. The tissue sections were subsequently deparaffinized using xylene, followed by conventional HE staining. Next, 5 random visual fields were selected from each section and the morphological changes in the tissues were assessed under an optical microscope (LX51, Olympus Optical Co., Ltd, Tokyo, Japan) [39, 41].

### TUNEL assay

The paraffin-embedded sections (6 μm) were dried at 62°C for 2 hours, deparaffinized using xylene, hydrated with alcohol, and rinsed with phosphate buffered saline (PBS). Then the sections were incubated with protease K at room temperature for 30 minutes and washed with PBS again. In order to inhibit the endogenous peroxidase activity, the sections were incubated with 3% H_2_O_2_ at room temperature for 20 minutes, washed with PBS, and incubated in equilibration buffer under room temperature for 20 minutes. After absorption of most of the equilibration buffer, the sections were incubated at 37°C for 1 hour with the addition of TdT buffer, washed with PBS, and incubated with serum at 37°C for 30 minutes, followed by nucleus staining with 4',6-Diamidino-2-Phenylindole (DAPI; Biohao Biotec Co., Ltd., Shanghai, China) for 20 minutes. After PBS washing, the sections were added with anti-fluorescence quenching agent and observed under a fluorescence microscope (XDY-1, Microscopes Inc., St. Louis, MI, USA). The number of stained cells observed in each image was manually measured using the AxioVision 4.2 software (Carl Zeiss, Thornwood, NY, USA) [40].

### HUVEC culture and treatment

HUVECs purchased from American Type Culture Collection (ATCC; Manassas, VA, USA) were cultured in endothelial cell medium containing 5% fetal bovine serum (FBS), 1% penicillin/streptomycin solution (Life Technologies, Inc., Paisley, UK), and 10% endothelial cell growth supplement (Sigma-Aldrich Chemical Company, St Louis, MO, USA). In accordance with the instructions of Lipofectamine 3000 (Invitrogen, Carlsbad, CA, USA), HUVECs were either exposed to 250 μM cobalt chloride (CoCl_2_) for 12 hours, or treated with 100 nM NC agomir, 100 nM miR-136-5p agomir, 100 nM NC antagomir, 100 nM miR-136-5p antagomir, 50 nM oe-NC, 50 nM oe-IL-6, or 50 nM oe-CRP and then subjected to exposure to 250 μM CoCl_2_ for 12 hours. The levels of IL-6 and CRP in the culture medium supernatant of HUVECs were detected using ELISA and turbidimetry standard method, independently [40, 42].

### *In vitro* assay based on the HUVEC damage model

HUVECs were exposed to 250 μM CoCl_2_ for about 12 hours to simulate the hypoxic/ischemic condition and induce a hypoxia damage model. Next, the apoptosis of HUVECs receiving different treatments was analyzed using flow cytometer (Accuri™ C6, BD Biosciences, San Diego, CA, USA) with propidium iodide (PI; eBioscience, CA, USA) and fluorescein isothiocyanate (FITC)-Anexin V staining methods [40, 42].

### Statistical analysis

All data were analyzed using the SPSS 21.0 statistical software (IBM Corp. Armonk, NY, USA). All data were tested for normal distribution and variance homogeneity. Data conforming to normal distribution were presented as mean ± standard deviation while the data with skewed distribution or heterogeneity of variance were expressed as interquartile range. Two-group comparisons were made using an unpaired *t*-test; multiple groups were compared using one-way analysis of variance (ANOVA), followed by the post hoc test. Two-group data with skewed distribution were analyzed using a non-parametric test (Wilcoxon signed ranks test). A *p* value < 0.05 was considered statistically significant.

### Ethics statement

This study protocol was approved by the Ethics Committee and Experimental Animal Ethics Committee of Qingdao Municipal Hospital. Written informed consent was obtained from all participants or their relatives prior to the enrollment in the study. Animal experiments were conducted in strict accordance with the principles of the *Guide for the Care and Use of Laboratory Animals* published by the US National Institutes of Health and all efforts were made to minimize the pain, suffering, and discomfort of the included animals.

## Supplementary Material

Supplementary Table 1
