# Supplementary material for: Downregulation of interleukin-6 and C-reactive protein underlies a novel inhibitory role of microRNA-136-5p in acute lower extremity deep vein thrombosis
Source: Aging (Albany NY). 2020 Nov 14;12(21):21076–90. doi: 10.18632/aging.103140 (PMC7695373; doi:10.18632/aging.103140)
Supplement: Supplementary Table 1 [file aging-12-103140-s001..docx]

**Supplementary Table 1. RIN value and purity of the RNA**

| Patient ID | RIN value | OD 260/280 ratio | OD 260/230 ratio |
| --- | --- | --- | --- |
| 1 | 8.62 ± 0.16 | 2.04 ± 0.04 | 2.11 ± 0.08 |
| 2 | 7.25 ± 0.13 | 2.18 ± 0.03 | 1.77 ± 0.07 |
| 3 | 7.98 ± 0.01 | 2.09 ± 0.04 | 1.96 ± 0.01 |
| 4 | 7.88 ± 0.23 | 2.13 ± 0.02 | 1.91 ± 0.07 |
| 5 | 7.78 ± 0.19 | 2.15 ± 0.04 | 1.86 ± 0.02 |
| 6 | 7.48 ± 0.09 | 2.17 ± 0.03 | 1.80 ± 0.08 |
| 7 | 7.13 ± 0.23 | 2.20 ± 0.05 | 1.76 ± 0.04 |
| 8 | 8.31 ± 0.15 | 2.06 ± 0.04 | 2.04 ± 0.08 |
| 9 | 7.54 ± 0.06 | 2.20 ± 0.02 | 1.81 ± 0.09 |
| 10 | 7.91 ± 0.09 | 2.10 ± 0.01 | 1.92 ± 0.06 |
| 11 | 8.39 ± 0.10 | 2.04 ± 0.02 | 2.07 ± 0.04 |
| 12 | 7.03 ± 0.01 | 2.20 ± 0.03 | 1.75 ± 0.07 |
| 13 | 8.91 ± 0.04 | 2.03 ± 0.05 | 2.16 ± 0.10 |
| 14 | 8.56 ± 0.16 | 2.07 ± 0.02 | 2.10 ± 0.05 |
| 15 | 8.45 ± 0.04 | 2.05 ± 0.03 | 2.08 ± 0.05 |
| 16 | 8.72 ± 0.10 | 2.01 ± 0.05 | 2.14 ± 0.08 |
| 17 | 8.50 ± 0.02 | 2.05 ± 0.03 | 2.10 ± 0.05 |
| 18 | 7.84 ± 0.07 | 2.14 ± 0.05 | 1.89 ± 0.09 |
| 19 | 8.75 ± 0.10 | 2.02 ± 0.03 | 2.15 ± 0.04 |
| 20 | 8.59 ± 0.13 | 2.04 ± 0.05 | 2.11 ± 0.08 |
| 21 | 8.22 ± 0.09 | 2.08 ± 0.04 | 2.00 ± 0.09 |
| 22 | 7.82 ± 0.04 | 2.18 ± 0.05 | 1.88 ± 0.02 |
| 23 | 8.35 ± 0.03 | 2.05 ± 0.02 | 2.06 ± 0.07 |
| 24 | 7.80 ± 0.08 | 2.15 ± 0.04 | 1.87 ± 0.07 |
| 25 | 7.73 ± 0.08 | 2.15 ± 0.01 | 1.84 ± 0.06 |
| 26 | 7.35 ± 0.16 | 2.16 ± 0.04 | 1.78 ± 0.10 |
| 27 | 8.97 ± 0.06 | 2.02 ± 0.04 | 2.20 ± 0.09 |
| 28 | 8.11 ± 0.08 | 2.12 ± 0.03 | 1.98 ± 0.04 |
| 29 | 7.86 ± 0.17 | 2.15 ± 0.05 | 1.91 ± 0.06 |
| 30 | 8.69 ± 0.21 | 2.00 ± 0.03 | 2.14 ± 0.06 |
| 31 | 8.56 ± 0.18 | 2.05 ± 0.02 | 2.10 ± 0.03 |
| 32 | 8.86 ± 0.18 | 2.01 ± 0.03 | 2.15 ± 0.03 |
| 33 | 7.54 ± 0.03 | 2.18 ± 0.04 | 1.81 ± 0.04 |
| 34 | 8.78 ± 0.19 | 2.00 ± 0.05 | 2.15 ± 0.07 |
| 35 | 7.83 ± 0.19 | 2.15 ± 0.01 | 1.89 ± 0.08 |
| 36 | 8.25 ± 0.22 | 2.12 ± 0.03 | 2.03 ± 0.07 |
| 37 | 7.50 ± 0.20 | 2.16 ± 0.03 | 1.80 ± 0.06 |
| 38 | 7.36 ± 0.01 | 2.16 ± 0.01 | 1.78 ± 0.04 |
| 39 | 7.75 ± 0.03 | 2.13 ± 0.04 | 1.85 ± 0.03 |
| 40 | 8.27 ± 0.24 | 2.03 ± 0.03 | 2.03 ± 0.06 |
| 41 | 7.60 ± 0.04 | 2.20 ± 0.04 | 1.83 ± 0.07 |
| 42 | 9.00 ± 0.02 | 2.01 ± 0.01 | 2.20 ± 0.05 |
| 43 | 7.97 ± 0.21 | 2.10 ± 0.01 | 1.95 ± 0.07 |
| 44 | 8.82 ± 0.14 | 2.02 ± 0.03 | 2.15 ± 0.01 |
| 45 | 8.77 ± 0.04 | 2.02 ± 0.04 | 2.15 ± 0.10 |
| 46 | 7.55 ± 0.12 | 2.18 ± 0.04 | 1.82 ± 0.06 |
| 47 | 8.50 ± 0.06 | 2.03 ± 0.04 | 2.09 ± 0.06 |
| 48 | 8.52 ± 0.18 | 2.05 ± 0.04 | 2.10 ± 0.03 |
| 49 | 7.54 ± 0.18 | 2.20 ± 0.03 | 1.80 ± 0.07 |
| 50 | 7.90 ± 0.05 | 2.08 ± 0.01 | 1.92 ± 0.07 |
| 51 | 8.70 ± 0.04 | 2.00 ± 0.01 | 2.14 ± 0.03 |
| 52 | 7.82 ± 0.02 | 2.13 ± 0.02 | 1.88 ± 0.05 |
| 53 | 7.89 ± 0.21 | 2.15 ± 0.05 | 1.92 ± 0.05 |
| 54 | 7.98 ± 0.06 | 2.08 ± 0.04 | 1.96 ± 0.08 |
| 55 | 7.76 ± 0.21 | 2.15 ± 0.03 | 1.85 ± 0.02 |
| 56 | 8.91 ± 0.08 | 2.00 ± 0.01 | 2.17 ± 0.10 |
| 57 | 7.62 ± 0.24 | 2.16 ± 0.04 | 1.84 ± 0.04 |
| 58 | 8.67 ± 0.06 | 2.00 ± 0.01 | 2.14 ± 0.09 |
| 59 | 8.93 ± 0.08 | 2.02 ± 0.05 | 2.19 ± 0.09 |
| 60 | 8.87 ± 0.18 | 2.00 ± 0.03 | 2.16 ± 0.04 |
| 61 | 8.29 ± 0.17 | 2.06 ± 0.03 | 2.04 ± 0.09 |
| 62 | 7.06 ± 0.15 | 2.19 ± 0.05 | 1.75 ± 0.09 |
| 63 | 8.66 ± 0.05 | 2.00 ± 0.03 | 2.13 ± 0.08 |
| 64 | 7.87 ± 0.06 | 2.13 ± 0.01 | 1.91 ± 0.08 |
| 65 | 8.58 ± 0.03 | 2.04 ± 0.01 | 2.11 ± 0.10 |
| 66 | 7.09 ± 0.09 | 2.20 ± 0.01 | 1.75 ± 0.01 |
| 67 | 7.62 ± 0.04 | 2.19 ± 0.05 | 1.83 ± 0.07 |
| 68 | 8.57 ± 0.08 | 2.03 ± 0.02 | 2.10 ± 0.10 |
| 69 | 7.65 ± 0.15 | 2.16 ± 0.01 | 1.84 ± 0.01 |
| 70 | 8.69 ± 0.19 | 2.00 ± 0.03 | 2.14 ± 0.04 |
| 71 | 8.64 ± 0.19 | 2.06 ± 0.03 | 2.12 ± 0.03 |
| 72 | 7.76 ± 0.01 | 2.13 ± 0.05 | 1.85 ± 0.06 |
| 73 | 7.86 ± 0.08 | 2.14 ± 0.05 | 1.91 ± 0.07 |
| 74 | 7.97 ± 0.22 | 2.12 ± 0.02 | 1.95 ± 0.03 |
| 75 | 8.41 ± 0.14 | 2.06 ± 0.02 | 2.07 ± 0.04 |
| 76 | 7.94 ± 0.20 | 2.07 ± 0.02 | 1.94 ± 0.05 |
| 77 | 8.39 ± 0.01 | 2.05 ± 0.04 | 2.06 ± 0.05 |
| 78 | 8.94 ± 0.09 | 2.01 ± 0.05 | 2.19 ± 0.06 |
| 79 | 7.44 ± 0.08 | 2.19 ± 0.03 | 1.80 ± 0.05 |
| 80 | 8.67 ± 0.02 | 2.00 ± 0.04 | 2.13 ± 0.06 |
| 81 | 8.37 ± 0.10 | 2.07 ± 0.02 | 2.06 ± 0.04 |
| 82 | 7.79 ± 0.13 | 2.13 ± 0.04 | 1.87 ± 0.05 |
| 83 | 7.01 ± 0.13 | 2.18 ± 0.05 | 1.75 ± 0.06 |
| 84 | 8.45 ± 0.10 | 2.06 ± 0.03 | 2.08 ± 0.02 |
| 85 | 7.32 ± 0.13 | 2.18 ± 0.02 | 1.78 ± 0.07 |
| 86 | 7.15 ± 0.05 | 2.20 ± 0.02 | 1.77 ± 0.01 |
| 87 | 7.58 ± 0.08 | 2.17 ± 0.02 | 1.82 ± 0.06 |
| 88 | 7.43 ± 0.17 | 2.18 ± 0.03 | 1.79 ± 0.05 |
| 89 | 8.40 ± 0.18 | 2.03 ± 0.01 | 2.07 ± 0.06 |
| 90 | 7.77 ± 0.10 | 2.15 ± 0.03 | 1.86 ± 0.07 |
| 91 | 8.23 ± 0.14 | 2.12 ± 0.05 | 2.00 ± 0.01 |
| 92 | 8.92 ± 0.10 | 2.01 ± 0.02 | 2.19 ± 0.01 |
| 93 | 7.50 ± 0.13 | 2.18 ± 0.02 | 1.80 ± 0.07 |
| 94 | 8.85 ± 0.13 | 2.02 ± 0.02 | 2.15 ± 0.06 |
| 95 | 8.64 ± 0.04 | 2.07 ± 0.01 | 2.12 ± 0.07 |
| 96 | 7.66 ± 0.17 | 2.14 ± 0.01 | 1.84 ± 0.01 |
| 97 | 8.19 ± 0.15 | 2.09 ± 0.02 | 1.99 ± 0.01 |
| 98 | 8.14 ± 0.14 | 2.11 ± 0.04 | 1.99 ± 0.08 |
| 99 | 8.34 ± 0.22 | 2.04 ± 0.03 | 2.05 ± 0.09 |
| 100 | 8.14 ± 0.04 | 2.12 ± 0.01 | 1.99 ± 0.07 |
| 101 | 7.95 ± 0.13 | 2.10 ± 0.04 | 1.95 ± 0.08 |
| 102 | 8.26 ± 0.12 | 2.09 ± 0.02 | 2.03 ± 0.08 |
| 103 | 8.23 ± 0.11 | 2.07 ± 0.01 | 2.00 ± 0.08 |
| 104 | 7.84 ± 0.17 | 2.12 ± 0.05 | 1.89 ± 0.07 |
| 105 | 7.70 ± 0.25 | 2.15 ± 0.03 | 1.84 ± 0.03 |
| 106 | 8.94 ± 0.03 | 2.03 ± 0.04 | 2.20 ± 0.07 |
| 107 | 7.86 ± 0.11 | 2.13 ± 0.05 | 1.91 ± 0.10 |
| 108 | 8.88 ± 0.23 | 2.07 ± 0.05 | 2.16 ± 0.07 |
| 109 | 8.64 ± 0.06 | 2.03 ± 0.03 | 2.12 ± 0.07 |
| 110 | 7.78 ± 0.08 | 2.15 ± 0.04 | 1.86 ± 0.01 |
| 111 | 8.28 ± 0.15 | 2.06 ± 0.02 | 2.03 ± 0.10 |
| 112 | 8.66 ± 0.14 | 2.02 ± 0.04 | 2.13 ± 0.08 |
| 113 | 8.42 ± 0.09 | 2.07 ± 0.01 | 2.08 ± 0.04 |
| 114 | 8.58 ± 0.20 | 2.03 ± 0.02 | 2.11 ± 0.10 |
| 115 | 8.51 ± 0.04 | 2.06 ± 0.03 | 2.10 ± 0.09 |
| 116 | 7.54 ± 0.02 | 2.16 ± 0.03 | 1.82 ± 0.02 |
| 117 | 8.13 ± 0.10 | 2.11 ± 0.02 | 1.98 ± 0.08 |
| 118 | 7.45 ± 0.15 | 2.18 ± 0.04 | 1.80 ± 0.04 |
| 119 | 8.17 ± 0.16 | 2.09 ± 0.03 | 1.99 ± 0.05 |
| 120 | 7.73 ± 0.15 | 2.15 ± 0.04 | 1.84 ± 0.01 |
| 121 | 7.43 ± 0.23 | 2.18 ± 0.05 | 1.79 ± 0.03 |
| 122 | 8.02 ± 0.20 | 2.13 ± 0.01 | 1.96 ± 0.08 |
| 123 | 8.73 ± 0.19 | 2.02 ± 0.01 | 2.15 ± 0.03 |
| 124 | 7.08 ± 0.24 | 2.20 ± 0.04 | 1.75 ± 0.07 |
| 125 | 8.10 ± 0.04 | 2.08 ± 0.01 | 1.98 ± 0.03 |
| 126 | 8.08 ± 0.11 | 2.09 ± 0.02 | 1.96 ± 0.10 |
| 127 | 7.45 ± 0.11 | 2.19 ± 0.05 | 1.80 ± 0.05 |
| 128 | 7.89 ± 0.11 | 2.09 ± 0.05 | 1.92 ± 0.07 |
| 129 | 7.10 ± 0.16 | 2.18 ± 0.04 | 1.75 ± 0.09 |
